# Protective Effect of *Panax notoginseng* Root Water Extract against Influenza A Virus Infection by Enhancing Antiviral Interferon-Mediated Immune Responses and Natural Killer Cell Activity

**DOI:** 10.3389/fimmu.2017.01542

**Published:** 2017-11-13

**Authors:** Jang-Gi Choi, Young-Hee Jin, Heeeun Lee, Tae Woo Oh, Nam-Hui Yim, Won-Kyung Cho, Jin Yeul Ma

**Affiliations:** ^1^Korean Medicine (KM) Application Center, Korea Institute of Oriental Medicine (KIOM), Daegu, South Korea

**Keywords:** herbal medicine, *Panax notoginseng* root, influenza A virus, H1N1, NK cell activity, splenocytes

## Abstract

Influenza is an acute respiratory illness caused by the influenza A virus, which causes economic losses and social disruption mainly by increasing hospitalization and mortality rates among the elderly and people with chronic diseases. Influenza vaccines are the most effective means of preventing seasonal influenza, but can be completely ineffective if there is an antigenic mismatch between the seasonal vaccine virus and the virus circulating in the community. In addition, influenza viruses resistant to antiviral drugs are emerging worldwide. Thus, there is an urgent need to develop new vaccines and antiviral drugs against these viruses. In this study, we conducted *in vitro* and *in vivo* analyses of the antiviral effect of *Panax notoginseng* root (PNR), which is used as an herbal medicine and nutritional supplement in Korea and China. We confirmed that PNR significantly prevented influenza virus infection in a concentration-dependent manner in mouse macrophages. In addition, PNR pretreatment inhibited viral protein (PB1, PB2, HA, NA, M1, PA, M2, and NP) and viral mRNA (NS1, HA, PB2, PA, NP, M1, and M2) expression. PNR pretreatment also increased the secretion of pro-inflammatory cytokines [tumor necrosis factor alpha and interleukin 6] and interferon (IFN)-beta and the phosphorylation of type-I IFN-related proteins (TANK-binding kinase 1, STAT1, and IRF3) *in vitro*. In mice exposed to the influenza A H1N1 virus, PNR treatment decreased mortality by 90% and prevented weight loss (by approximately 10%) compared with the findings in untreated animals. In addition, splenocytes from PNR-administered mice displayed significantly enhanced natural killer (NK) cell activity against YAC-1 cells. Taking these findings together, PNR stimulates an antiviral response in murine macrophages and mice that protects against viral infection, which may be attributable to its ability to stimulate NK cell activity. Further investigations are needed to reveal the molecular mechanisms underlying the protective effects of PNR and its components against influenza virus A infection.

## Introduction

Influenza, an acute viral respiratory illness caused by the influenza virus, is highly contagious and causes global epidemics every year (1–3). Infants and the elderly are particularly susceptible to this disease, which is associated with severe economic and social losses (4–6). Globally, approximately one billion people contract influenza each year, and the disease is estimated to cause approximately 300,000–500,000 deaths annually (7, 8).

Currently, vaccines and antiviral agents are mainly used to suppress influenza infection (9). For seasonal influenza caused by antigenic drift, developing effective vaccines is possible to some extent. However, it is difficult to predict when outbreaks of new influenza viruses caused by antigenic shift will occur. Moreover, influenza vaccines have recently become ineffective for controlling new infections (10). Therefore, antiviral agents have an important role in disease control together with vaccines, as they can prevent further spread of disease and treat currently infected patients (11).

Two groups of antiviral agents, namely, neuraminidase (NA) inhibitors (peramivir, oseltamivir, laninamivir, and zanamivir) and M2 ion channel inhibitors (rimantadine and amantadine), can be used to treat and prevent influenza virus infection (11, 12). However, long-term or repeated use of antiviral agents promotes the emergence of drug-resistant influenza viruses (12–14). This has led to a situation in which most virus strains are resistant to M2 ion channel inhibitors, so now only NA inhibitors are used to treat influenza. Unfortunately, influenza viruses resistant to NA inhibitors are also emerging globally. Thus, there is an urgent need for new antiviral agents to prevent influenza pandemics. For this reason, much attention has been devoted to identifying natural compounds with activity against influenza. For example, extracts from plants such as *Salacia reticulata* (15), *Ribes nigrum folium* (16), *Cryptoporus volvatus* (17), and *Psoraleae semen* (18) displayed potent antiviral activity against influenza A virus.

*Panax notoginseng* root (PNR), also known as sanchi or Radix Notoginseng, is a well-known nutritional supplement, health food additive, and traditional medicine from the genus *Panax* (Araliaceae) (19, 20). It has been used for thousands of years as a hemostatic agent to regulate intracerebral hemorrhage in Korea and China (20). Currently, PNR is widely used to treat cardiovascular disease due to its vasodilatory and antihypertensive effects (20). According to recent research, PNR exhibits antiplatelet (21), anticoagulant (21), antihypertensive, antimicrobial (22), antitumor (23–26), antioxidant (23), anti-inflammatory (27), anticoagulation (27), antiatherosclerotic (27), and neuroprotective activities (28). However, it has not been established whether PNR exhibits activity against influenza virus.

The innate immune system consists of a range of components including type-I interferon (IFN) and pro-inflammatory cytokines, and is the first line of defense against viral infection (29). Another component of the innate immune system, natural killer (NK) cells, as well as the adaptive immune system cells macrophages, have been reported to inhibit virus replication and protect against cancer (15, 18). NK cells are known to provide the first line of early defense against viral infections (30–32). Studies have shown that inhibition of NK cell function and depletion of NK cells in mice can lead to morbidity and mortality as well as delayed clearance of influenza virus infection (33–36). The role of NK cells in this context involves cytokine secretion and cytotoxicity against infected target cells (31). Different types of IFNs and pro-inflammatory cytokines have various effects in activating NK cells and selectively killing virus-infected cells (37–39). Thus, the generation of type-I IFNs and pro-inflammatory cytokines plays an important role in regulating the immune system for NK cell activation and cytotoxicity (39–41). Given this background, immunomodulatory agents can play an important role as they are known to increase host immunity (enhance NK cell activity) and resistance to viral infection or cancer (42). Through nutritional supplements, it may be possible to strengthen the antiviral host defense response, which could be effective in the body’s resistance to influenza. Accordingly, many researchers have searched for herbal medicines or natural products with immunomodulatory activities to overcome influenza virus infection (15, 18, 43, 44). We investigated PNR-inducing signal molecules that activate antiviral mediators such as type-I IFNs, pro-inflammatory cytokines, and NK cell activity.

In this study, we investigated whether PNR has the ability to inhibit influenza virus infection *in vitro* and *in vivo*. We first examined its potential to inhibit influenza virus replication and the mechanisms of action of this *in vitro*, and then determined whether PNR could protect mice from lethal challenge with influenza virus. Our results showed that PNR significantly prevented influenza virus infection in murine macrophage cells. In addition, PNR pretreatment inhibited the expression of viral protein and viral mRNA. PNR pretreatment also increased the secretion of pro-inflammatory cytokines and the phosphorylation of type-I IFN-related proteins *in vitro*. Moreover, in mice exposed to the influenza A H1N1 virus, PNR treatment decreased mortality and prevented weight loss compared with the findings in untreated animals. Furthermore, splenocytes from PNR-administered mice displayed significantly enhanced NK cell activity against YAC-1 cells.

## Materials and Methods

### PNR Preparation

*Panax notoginseng* root was purchased from Yeongcheon Oriental Herbal Market (Yeongcheon, South Korea) and stored at the KM-Application Center herbarium (registration number, #76) KIOM, after its identity had been confirmed by Prof. Ki Hwan Bae (Chungnam National University, Daejeon, South Korea). For preparation, dried PNR (50 g) was immersed in distilled water (1 L) and then heat-extracted at 115°C for 3 h. After being filtered through a sieve (150 µm), PNR was freeze-dried and stored in desiccators at 4°C until further use.

### Chemical Reagents and Chromatographic Conditions

High-performance liquid chromatography-grade acetonitrile was purchased from Merck KGaA (Darmstadt, Germany). Ultrapure water (UW) was obtained using a Puris-Evo UP Water system with Evo-UP Dio VFT and Evo-ROP Dico20 (Mirae ST Co., Ltd., Anyang, Gyeonggi-do, South Korea). UW was prepared with resistivity of 18.2 MΩ cm^−1^ (Puris, Esse-UP Water system; Mirae St. Co., Anyang, South Korea). Notoginsenoside R1 was purchased from NIKOM (National Development Institute of Korean Medicine, Gyeongsan, Gyeongsangbuk-do, South Korea), and ginsenosides Rg1, Rb1, and Rd were purchased from ChemFaces (Hubei, China). The purity of these standards exceeded 98.0%.

Chromatographic analysis was performed using a Hitachi HPLC system (Hitachi Co., Tokyo, Japan) consisting of a pump (L-2130), autosampler (L-2200), column oven (L-2350), diode array UV/VIS detector (L-2455), and an Alltech ELSD 3300 detector (Alltech, Deerfield, IL, USA). A Thermo Acclaim C18 column (Thermo Fisher Scientific Inc., Waltham, MA, USA) was maintained at 40°C for sample analysis. Data acquisition was performed using EZChrom Elite software (Hitachi). The mobile phase consisted of water (A) and water plus acetonitrile (B) at a flow rate of 1.5 mL/min with the following gradient flow: 18–19% B at 0–30 min; 19–31% B at 30–40 min; and 31–56% B at 40–60 min. The autosampler injection volume was 10 µL and the total run time was 60 min.

### Preparation of Standard Solutions and Samples

*Panax notoginseng* root samples were accurately weighed (12.5 mg), immersed in 1 mL of 100% methanol, and extracted *via* ultrasonication for 30 min. The standard stock solutions were prepared by dissolution in 100% methanol (1 mg/mL). All working solutions were filtered through a 0.2-mm syringe membrane filter (Whatman Ltd., Maidstone, UK) before injection into the HPLC system.

### Cells and Viruses

RAW 264.7, YAC-1, and Madin–Darby canine kidney (MDCK) cells were obtained from the American Type Culture Collection. The MDCK cells were maintained in DMEM containing 10% (v/v) heat-inactivated fetal bovine serum (FBS) and antibiotics (penicillin and streptomycin) at 37°C and 5% CO_2_ (18). RAW 264.7 and YAC-1 were maintained in RPMI 1640 containing 10% (v/v) heat-inactivated FBS and antibiotics (penicillin and streptomycin) at 37°C and 5% CO_2_.

Influenza A strains [green fluorescent protein (GFP)-tagged A/PR/8/34 (A/PR/8/34-GFP) and Puerto Rico/8/34 (A/PR/8/34)] were obtained from Prof. Jong-Soo Lee (Chungnam National University, Daejeon, South Korea). KBPV-VR-32 (H3N2) was purchased from the Korea Bank for Pathogenic Viruses. All three strains were propagated in allantoic fluid from 10-day-old chicken embryos (18, 45) and viral titers were determined as described previously (18, 45).

### Reagents and Antibodies

Lipopolysaccharide, IFN-β (recombinant mouse), and oseltamivir phosphate were obtained from Sigma-Aldrich (St. Louis, MO, USA). Anti-IRF3, anti-STAT1, anti-TBK1, anti-phospho-IRF3, anti-phospho-STAT1, and anti-phospho-TBK1 antibodies were purchased from Cell Signaling Technology (Boston, MA, USA), and antibodies targeting influenza proteins (NP, PA, HA, PB1, PB2, M1, M2, and NA) were obtained from GeneTex (San Antonio, TX, USA). Anti-β-actin was acquired from Santa Cruz Biotechnology (Santa Cruz, CA, USA).

### Cell Viability

For trypan blue exclusion analysis, MDCK, YAC-1, and RAW 264.7 cells were seeded into 24-well plates at a density of 1 × 10^5^ cells/well and cultured overnight before PNR treatment. PNR was added to the wells at various concentrations (0, 6.25, 12.5, 25, 50, 100, 200, or 400 µg/mL). After 24 h of incubation, cells were collected and stained with trypan blue dye for 5 min at room temperature. The cell suspension was then applied to an Invitrogen Countess II FL Automated Cell Counter and the cells stained with trypan blue were regarded as dead cells.

### Animal Studies

This study was carried out in accordance with the guidelines of the Institutional Animal Care and Use Committee (IACUC) of the Laboratory Animal Center of Daegu-Gyeongbuk Medical Innovation Foundation (DGMIF). Animal studies were approved by the IACUC of the Laboratory Animal Center of DGMIF under approval number DGMIF-17031401-01.

BALB/c mice (females, 5 weeks old) from Orient Bio Inc. (Seongnam, South Korea) were acclimated for at least 1 week under standard housing conditions at DGMIF and provided a standard rodent chow diet and water *ad libitum*.

For oral inoculation of PNR and influenza A virus challenge, mice were separated into three experimental sets containing three groups of 10 mice per set [phosphate-buffered saline (PBS), PNR (100 mg/kg) with virus infection, and PBS with virus infection]. Mice in the latter two groups were orally administered 100 mg/kg PNR at a total volume of 200 µL once daily for 7 days before infection.

Mice were infected intranasally with five times the 50% mouse lethal dose (LD_50_) of A/PR/8/34 in 20 µL of PBS. Body weights and survival were monitored for 6 days after infection (dpi) at fixed time points. At 5 dpi, three mice from each group were randomly sacrificed to measure lung histopathology, and the remaining mice were used to examine survival. Histopathologically, lung tissues were immediately fixed in paraffin-embedded neutral buffer containing 10% formalin, sectioned to 4- to 6-µm thickness using a microtome device, mounted on a slide, stained with eosin, and examined under an optical microscope as described previously (46).

NK activity was measured using slightly modified versions of a previously described flow cytometric assay and lactate dehydrogenase (LDH) assay (15, 47). BALB/c mice (6 weeks old, female) were orally administered 200 µL of 100 mg/kg PNR or PBS once daily for 7 days, after which splenocytes were isolated. YAC-1 cells were stained with CFSE (eBioscience, San Diego, CA, USA), according to the manufacturer’s protocol.

Splenocytes were used as effector cells and YAC-1 cells were used as target cells. Splenocytes were added to 200 µL of culture medium in the wells of a 96-well culture plate containing 1 × 10^4^ CFSE-labeled YAC-1 cells at ratios of 5:1, 10:1, and 50:1. After incubation for 4 h, the amount of LDH liberated from the target cells *via* spleen NK cell activity was measured using an LDH Cytotoxicity Assay Kit II (Abcam, Cambridge, UK), as per the manufacturer’s instructions. After incubation for 24 h, the percentage of spontaneously lysed CFSE-labeled YAC-1 cells was determined by flow cytometry.

### Enzyme-Linked Immunosorbent Assay (ELISA)

Interleukin (IL)-6, IL-1β, and tumor necrosis factor (TNF)-α (mouse) levels in culture supernatants were determined using ELISA antibody kits (eBioscience), according to the supplier’s instructions (18).

### Viral Replication Inhibition Assay

The inhibition of viral replication was assayed as previously described (18). Briefly, RAW 264.7 cells were cultured in six-well plates (1 × 10^6^ cells/well) for 12 h. Cells were then exposed to medium (RPMI, negative control), 1,000 U of recombinant mouse IFN-β (positive control), or 10 or 100 µg/mL of PNR. After 12 h, the cells were infected with PR8/34-GFP [multiplicity of infection (MOI) = 1]. GFP expression was observed under a microscope after 24 h of viral infection, and cell death was measured by the MTS assay (18). The viral yield reduction assay was performed as previously described (18).

### Neuraminidase Inhibition (NI) Assay

The NI assay was performed using an NA-Fluor™ Influenza Neuraminidase Assay Kit (Applied Biosystems, Foster City, CA, USA), as per the manufacturer’s instructions (45).

### Quantitative Real-time Polymerase Chain Reaction (qRT-PCR)

Total RNA was extracted from cell lysates using an RNeasy mini kit (Qiagen, Hilden, Germany), according to the supplier’s instructions (18, 45). qRT-PCR was performed using AccuPower^®^ 2× Greenstar qPCR Master Mix (Bioneer, Daejeon, South Korea) and a CFX96 Touch Real-Time PCR System (Bio-Rad, Hercules, CA, USA), according to the suppliers’ instructions (18, 45). The sense and antisense primer sequences are listed in Table 1.

**Table 1:** Primer sequences for quantitative real-time polymerase chain reaction.

Name	Orientation	Primer sequences 5′–3′ orientation
β-Actin	Forward	AGGTGTGCACCTTTTATTGGTCTCAA
	Reverse	TGTATGAAGGTTTGGTCTCCCT
M1	Forward	GCATCGGTCTCATAGGCAAATG
	Reverse	CCTCTGCTGCTTGCTCACTC
M2	Forward	GAAAGGAGGGCCTTCTACGG
	Reverse	TCGTCAGCATCCACAGCAC
NP	Forward	GAATGGTGCTCTCTGCTTTTGA
	Reverse	TCCACTTTCCGTTTACTCTCCTG
PA	Forward	AAGTGCCATAGGCCAGGTTTC
	Reverse	CCTCATCTCCATTCCCCATTTC
PB2	Forward	GGTGCTTACGGGCAATCTTC
	Reverse	TGTTCGTCTCTCCCACTCACTATC
NS1	Forward	GCGATGCCCCATTCCTTG
	Reverse	ATCCGCTCCACTATCTGCTTTC-
HA	Forward	TTGCTAAAACCCGGAGACAC
	Reverse	CCTGACGTATTTGGGCACT

### Immunofluorescence Staining

Immunofluorescence staining was performed as previously described (18). RAW 264.7 cells (1 × 10^5^) grown on four-well tissue culture slides were incubated at 37°C for 12 h. PNR (100 µg/mL) and IFN-β (1,000 U) were added to the cells, which were cultured in a CO_2_ incubator at 37°C for 12 h. Then, the medium was discarded and the cells were washed with PBS and infected with H1N1 (MOI = 1) for 2 h (18). Next, cells were cultured in a CO_2_ incubator at 37°C for 24 h. Cells were then washed (with PBS, three times) and fixed with paraformaldehyde (4%) and 1% Triton X-100 for 30 min at room temperature (18). After blocking, the fixed cells were incubated overnight with M2-specific antibody, washed three times with TBS, and incubated with Alexa Fluor 568 goat anti-rabbit IgG antibody (1:1,000; Life Technologies, Eugene, OR, USA). Next, the cells were incubated with DAPI for 10 min and observed under a fluorescence microscope (18).

### Western Blotting

RAW 264.7 cells (1 × 10^6^ cells/well) were harvested at the indicated times. Equal amounts of protein lysate in RIPA buffer were separated by 12% SDS-PAGE (18, 45). After transfer, PVDF membranes were incubated at room temperature for 1 h with primary antibodies (1:1,000) followed by HRP-conjugated secondary antibodies (1:2,000) at room temperature for 1 h. Protein bands were detected using enhanced chemiluminescence reagent and a ChemiDoc imaging system (45). The presented data are representative of at least three independent experiments. Densitometric analyses were performed using ImageJ software.

### Statistical Analysis

Data are expressed as mean ± SEM. The statistical significance of differences in the mean values between the treatment and control groups were determined using Chi-squared test and Bonferroni correction. For analysis of three or more groups, ANOVA was performed with Bonferroni’s posttest. Survival analysis was performed by the Kaplan–Meier method and the statistical significance of differences was determined by the log-rank test. Analyses were performed using GraphPad PRISM software^®^ Version 5.02 (GraphPad, La Jolla, CA, USA). *P* < 0.05 was considered to denote statistical significance.

## Results

### HPLC Analysis of PNR

**Figure 1:**
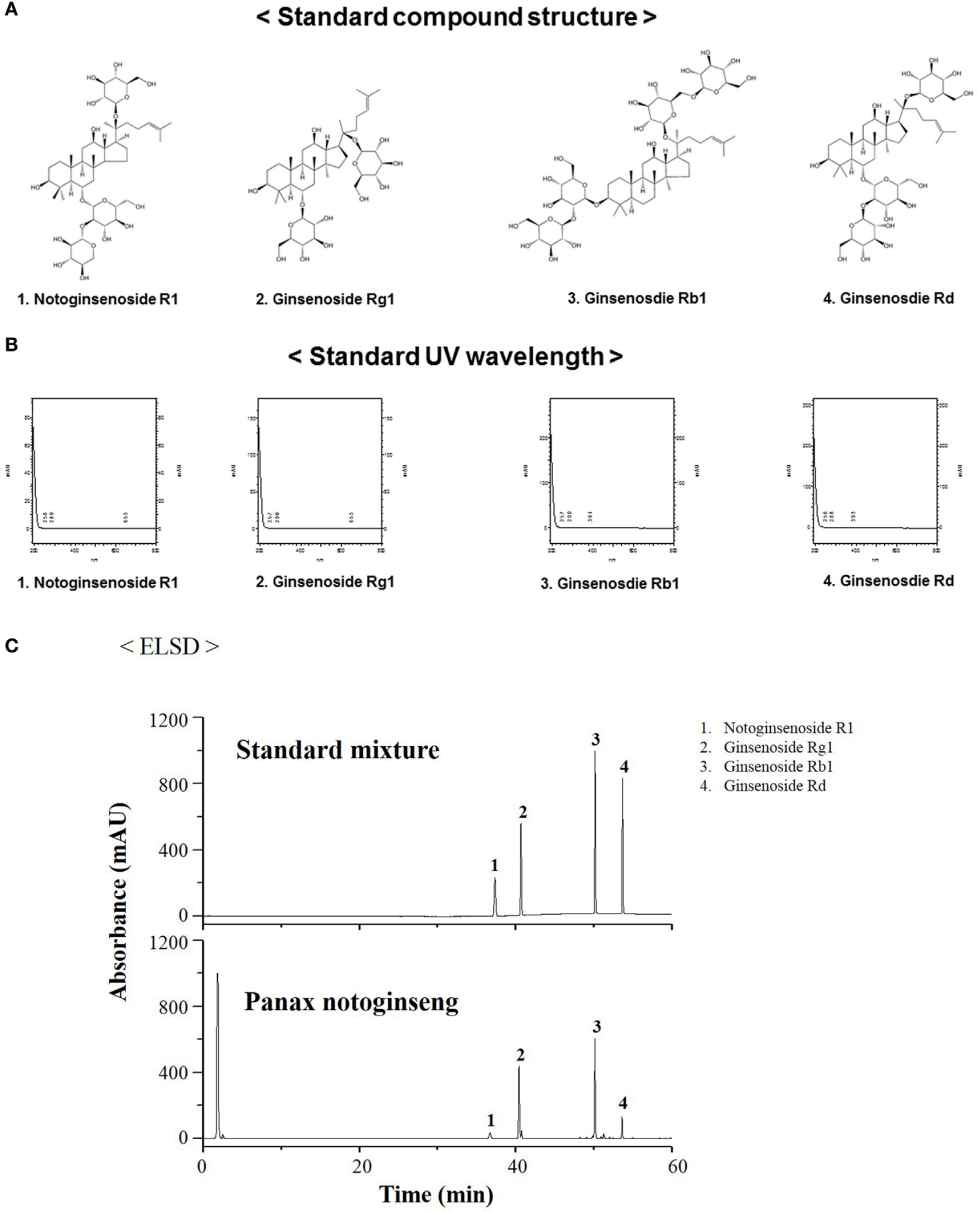
Measurement of the representative components of *Panax notoginseng* root (PNR) by high-performance liquid chromatography. Standard compound structure **(A)**, standard UV wavelength **(B)**, notoginsenoside R1 (1), ginsenoside Rg1 (2), ginsenoside Rb1 (3), and ginsenoside Rd (4) in the standard mixture **(B)**, and PNR **(C)** at a wavelength of 245 nm.

Representative HPLC chromatograms of the standard mixture and extracts of PNR samples are presented in Figure 1. Peaks were observed for four compounds, namely, notoginsenoside R1 (36.17 min), ginsenoside Rg1 (40.45 min), ginsenoside Rb1 (50.16 min), and ginsenoside Rd (53.633 min). PNR ingredients were recognized; their peaks were identified by comparing their retention times with those of notoginsenoside R1, ginsenoside Rg1, ginsenoside Rb1, and ginsenoside Rd, followed by adding the standard solution to PNR under the same conditions and eluting (48, 49).

### Effects of PNR on Cell Cytotoxicity

**Figure 2:**
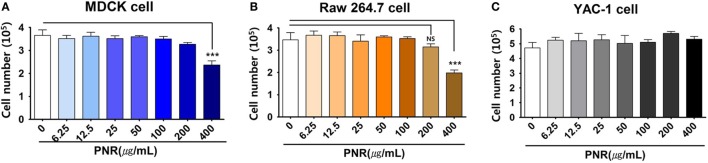
Determination of the cytotoxicity of *Panax notoginseng* (PNR) water extract in MDCK **(A)**, RAW 264.7 **(B)** and YAC-1 **(C)** cells. The viability of RAW 264.7, YAC-1, and MDCK cells was assessed using trypan blue exclusion analysis after 24 h of treatment with PNR at the indicated concentrations. Data represent the mean ± SEM of three independent experiments. Statistical significance between values obtained for PNR-treated and PNR-untreated cells were determined using Chi-square test, ***P < 0.001; n.s., not significant.

To determine the optimal concentration of PNR that produced antiviral activity with minimal cytotoxicity, we investigated the cytotoxicity of PNR using the trypan blue analysis after treating MDCK, RAW 264.7, and YAC-1 cells with PNR for 24 h. The results illustrated that PNR is not cytotoxic in either cell line at concentrations of ≤200 μg/mL; however, at a concentration of 400 µg/mL, PNR showed cytotoxicity in MDCK and RAW 264.7 cells (Figures 2A–C). Therefore, subsequent experiments were performed using PNR concentrations of <100 μg/mL.

### PNR Induced the Secretion of Pro-inflammatory and IFN-β Cytokines and the Activation of the Type-I IFN Signaling Pathway in Murine Macrophages

**Figure 3:**
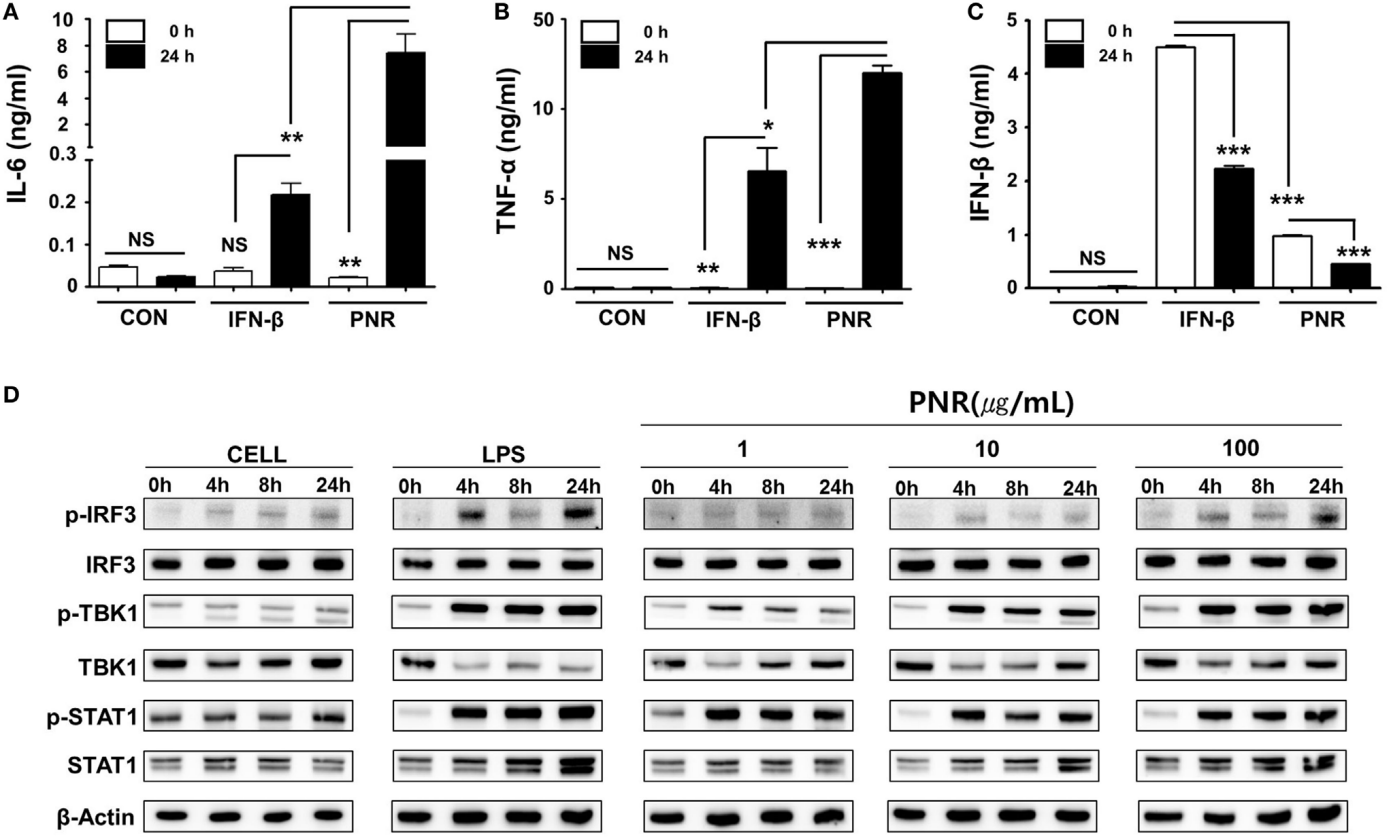
Induction of pro-inflammatory cytokines and activation of type-I interferon (IFN) by *Panax notoginseng* root (PNR) *in vitro*. **(A–C)** RAW 264.7 cells were treated with DMEM containing 10% fetal bovine serum (FBS) alone, 1,000 U/mL recombinant mouse IFN-β, or 100 µg/mL PNR and incubated at 37°C and 5% CO_2_. Supernatant from each group was harvested at 0 and 24 h and clarified by centrifugation at 2,500*g* for 10 min at 4°C. Clarified supernatants were dispensed into murine interleukin (IL)-6, tumor necrosis factor (TNF)-α, and IFN-β capture antibody-coated enzyme-linked immunosorbent assay plates to measure cytokine secretion. The test was performed in duplicate. **(D)** Western blotting was performed using the whole-cell lysates of macrophage-type cells treated with or without PNR (1, 10, and 100 µg/mL) to assess the expression of the nonphosphorylated and phosphorylated forms of IRF3, TANK-binding kinase 1 (TBK1), STAT1, and β-actin over time. Similar results were obtained, and the experiment was performed three times independently. Bar graph (mean ± SEM) statistics were determined using two-way ANOVA with Bonferroni’s correction (posttest), ***P < 0.001; **P < 0.01; *P < 0.05. n.s., not significant.

Type-I IFN and pro-inflammatory cytokines play an important role in inducing antiviral responses and immunoregulatory activities (50). Thus, we investigated effects of PNR on the secretion of pro-inflammatory cytokines, namely, IL-6, TNF-α, and IFN-β. Experiments were performed by treatment with PNR, IFN-β, or medium alone (CON) in RAW 264.7 cell cultures. After 24 h of incubation, IL-6 production levels were measured and the results (Figure 3A) show that 100 µg/mL of PNR induces production of IL-6 (7.45 ± 1.392 ng/mL). However, the cells treated with media alone did not produce significant levels of cytokines (0.023 ± 0.0017 ng/mL). In addition, Figure 3B show that PNR induced a significant increase in the production of TNF-α when used at 100 µg/mL of PNR (11.984 ± 4.245 ng/mL), and cells treated with medium alone released very low levels of TNF-a (0.019 ± 0.0017 ng/mL). Similarly, Figure 3C show that, the IFN-β secretion levels increased significantly with PNR (100 µg/mL, 0.451 ± 0.907 ng/mL). These results show that pro-inflammatory cytokines (IL-6 and TNF-α) and IFN-β can be induced by PNR, which can mediate the antiviral status in murine microphage cells. In addition, the antiviral response of PNR may be associated with innate immune responses through the expression of type-I IFN and pro-inflammatory cytokines.

To correlate the above observations with the IFN signaling pathway, we investigated the effect of PNR on type-I IFN-related protein phosphorylation. PNR-treated cells were used for Western blotting to analyze the expression of nonphosphorylated and phosphorylated forms of IRF3, STAT1, and TANK-binding kinase 1 (TBK1). IRF3, STAT1, and TBK1 phosphorylation was increased following treatment with PNR (100 µg/mL) in RAW 264.7 cells (Figure 3D) compared with the findings in untreated cells. The phosphorylation of IRF3 represents the translocation of IRF3 molecules into the nucleus and the initiation of transcription of type-I IFNs (51). This induces type-I IFN to bind to a component of the JAK/STAT pathway, leading to the phosphorylation of STAT1 and transcriptional activation of the IFN-stimulated genes (51). Taking all of these findings together, it is suggested that PNR can induce an antiviral status of RAW264.7 cells by modulating the IFN signaling pathway, which can inhibit viral replication.

### PNR Inhibited Influenza A Virus Infection in RAW 264.7 Cells

**Figure 4:**
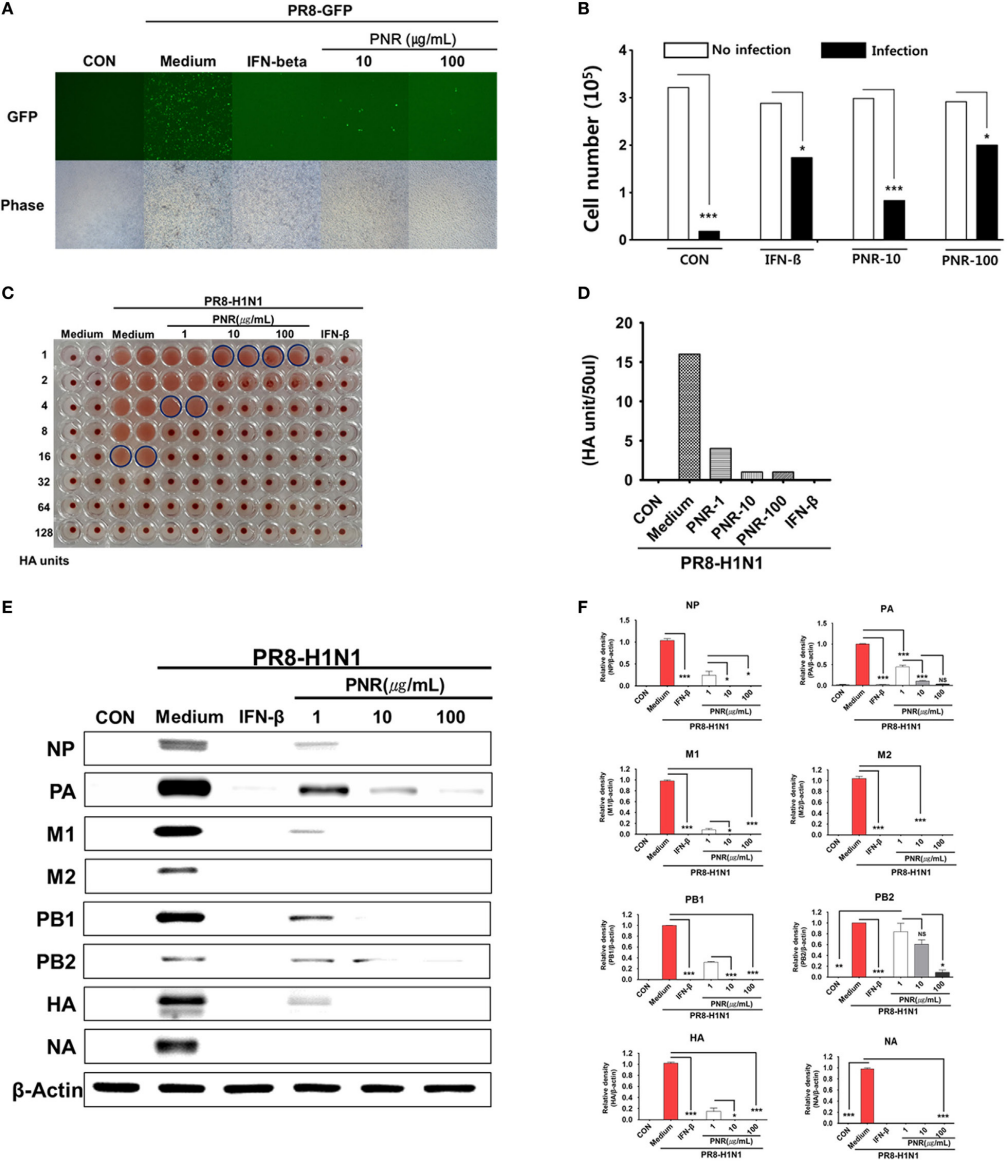
Antiviral activities of *Panax notoginseng* root (PNR) on the influenza A/PR/8/34-GFP virus in RAW 264.7 cells. **(A)** RAW 264.7 cells were treated with PNR (10 and 100 µg/mL) prior to A/PR/8/34-GFP infection, and cells were incubated with medium alone, 10 or 100 µg/mL PNR, or 1,000 U/mL interferon (IFN)-β 12 h prior to infection with A/PR/8/34-GFP (multiplicity of infection = 1). Images of GFP expression were obtained 24 h after virus infection. **(B)** Cell counts and viabilities were determined 24 h after virus infection using the trypan blue exclusion analysis. Statistical significance between values obtained for virus infection and no virus infection cells was determined using Chi-square test and Bonferroni’s correction (post-test), ***P < 0.001; *P < 0.05; n.s., not significant. **(C,D)** Viruses were titrated from the supernatant *via* the hemagglutination assay. **(E,F)** RAW 264.7 cells were treated with PNR (1, 10, or 100 µg/mL), IFN-β (1,000 U/mL), or medium alone (negative control). Influenza A/PR/8/34 A virus protein levels (NP, PA, M1, M2, PB1, PB2, HA, and NA) in cell lysates were analyzed by Western blotting, and β-actin expression was analyzed as an internal control. Similar results were obtained, and the experiment was performed three times independently. Bar graph (mean ± SEM) statistics were determined using two-way ANOVA with Bonferroni’s correction (post-test), ***P < 0.001; **P < 0.01; *P < 0.05. n.s., not significant.

To evaluate the anti-influenza A virus effects of PNR, we used GFP-expressing viruses as previously described (18, 45). Pretreatment of RAW 264.7 cells infected with A/PR8/34-GFP (MOI = 1) with PNR resulted in a marked reduction in GFP expression in a concentration-dependent manner (Figure 4A), and virus-induced death was also concentration-dependently suppressed in pretreated cells (Figure 4B). We also confirmed that, compared with the supernatant titer of A/PR8/34-GFP-infected cells that were untreated [16 hemagglutination units (HAUs)], those of A/PR8/34-GFP- and PNR/A/PR8/34-GFP-infected cells treated with 1 (4 HAUs), 10, or 100 µg/mL of PNR (1 HAU) or IFN-β (0 HAU) were significantly decreased (Figures 4C,D). These results suggested that PNR inhibited A/PR8/34-induced GFP expression and cell death in RAW 264.7 cells.

We investigated whether PNR inhibited A/PR8/34 viral protein (NP, PA, M1, M2, PB1, PB2, HA, and NA) expression using Western blotting in RAW 264.7 cells pretreated with PNR (1, 10, or 100 µg/mL). The results illustrated that PNR significantly inhibited the expression of all studied A/PR8/34 viral proteins, with the exception of that of PA, in a concentration-dependent manner (Figures 4E,F).

**Figure 5:**
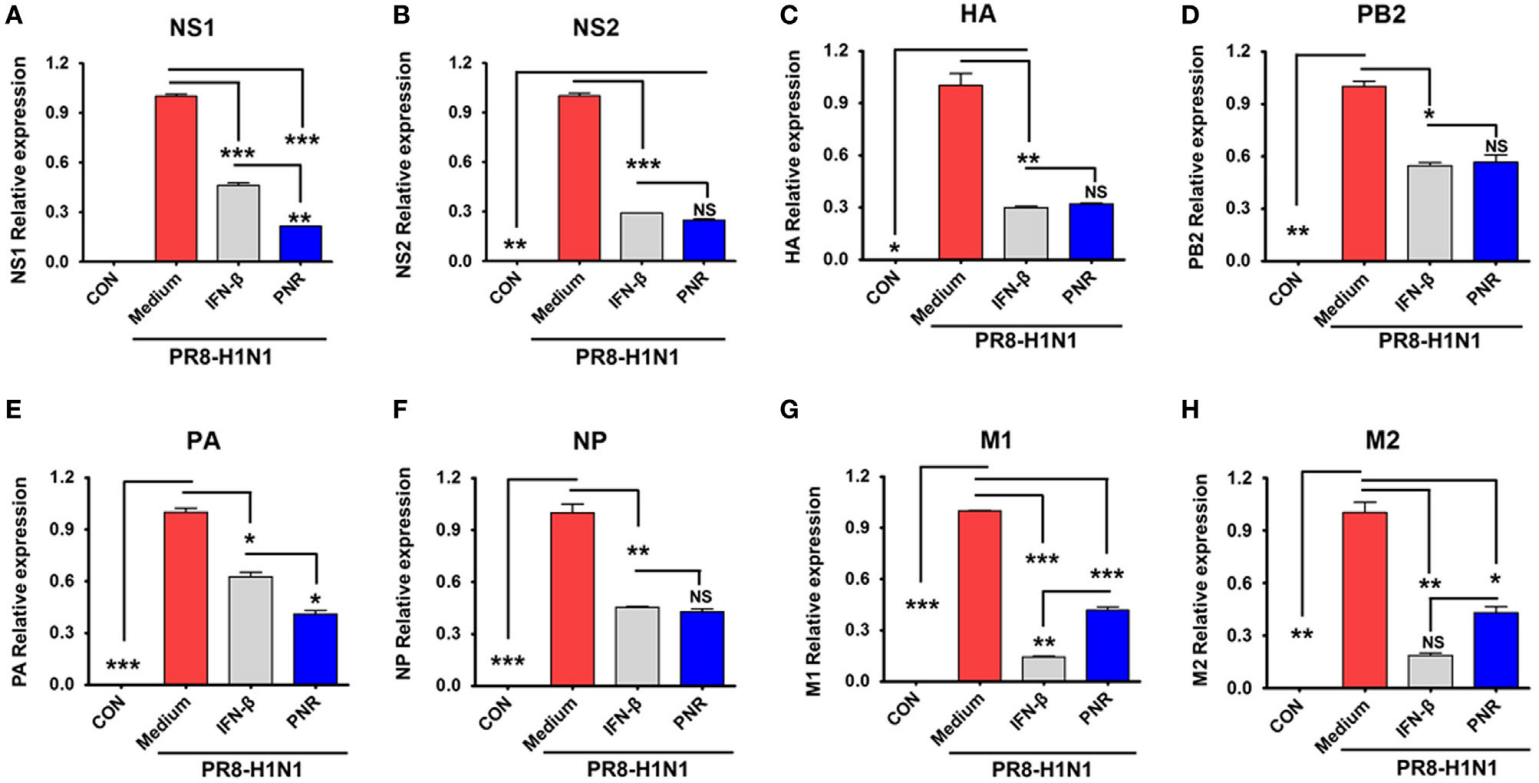
Effect of constituents from *Panax notoginseng* root (PNR) on influenza A/PR/8/34 mRNA synthesis. Pretreatment with PNR (100 µg/mL) and interferon (IFN)-β (1,000 U/mL) on influenza A/PR/8/34 (multiplicity of infection = 1)-infected RAW 264.7 cells and the relative mRNA levels of influenza A/PR/8/34 NS1 **(A)**, NS2 **(B)**, HA **(C)**, PB2 **(D)**, PA **(E)**, NP **(F)**, M1 **(G)** and M2 **(H)** were analyzed by quantitative real-time polymerase chain reaction and normalized to β-actin levels. Data are presented as the mean ± SD of three independent experiments. Bar graph (mean ± SEM) statistics were determined using two-way ANOVA with Bonferroni’s correction (post-test), ***P < 0.001; **P < 0.01; *P < 0.05. n.s., not significant.

We also investigated whether PNR decreased A/PR8/34 viral mRNA (NS1, HA, PB2, PA, NP, M1, and M2) synthesis using qRT-PCR. NS1 and PA mRNA levels (Figures 4A,E) were significantly decreased by PNR pretreatment (100 µg/mL) in RAW 264.7 cells compared with the findings in the IFN-β and virus-only groups. In addition, NS1, HA, PB2, NP, M1, and M2 mRNA synthesis was significantly inhibited by PNR pretreatment (100 µg/mL) relative to the findings in the virus-only groups (Figure 5).

**Figure 6:**
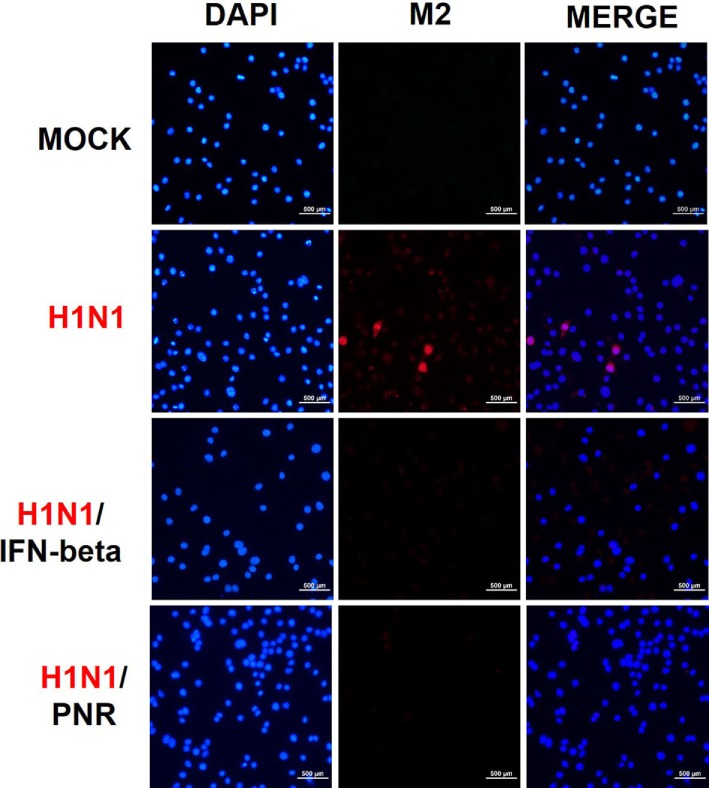
*Panax notoginseng* root (PNR) reduced the expression of the M2 protein of influenza A/PR/8/34 (H1N1) virus in infected RAW 264.7 cells. Immunofluorescence analysis illustrated the level of influenza A M2 protein in RAW 264.7 cells. Cells were treated with PNR (100 µg/mL) or interferon (IFN)-β (1,000 U/mL) after influenza A virus infection. An influenza A virus M2-specific antibody was used to observe the protein in RAW 264.7 cells *via* fluorescence microscopy. Cells were also stained with DAPI, and the merged image shows the cytoplasmic location of M2 (red).

We further examined the effects of 100 µg/mL of PNR on M2 protein expression *via* immunofluorescent analysis after 24 h of exposure to A/PR/8/34. M2 protein expression was significantly suppressed in RAW 264.7 cells treated with PNR or IFN-β (Figure 6).

### Effects of PNR on NA Activity

**Figure 7:**
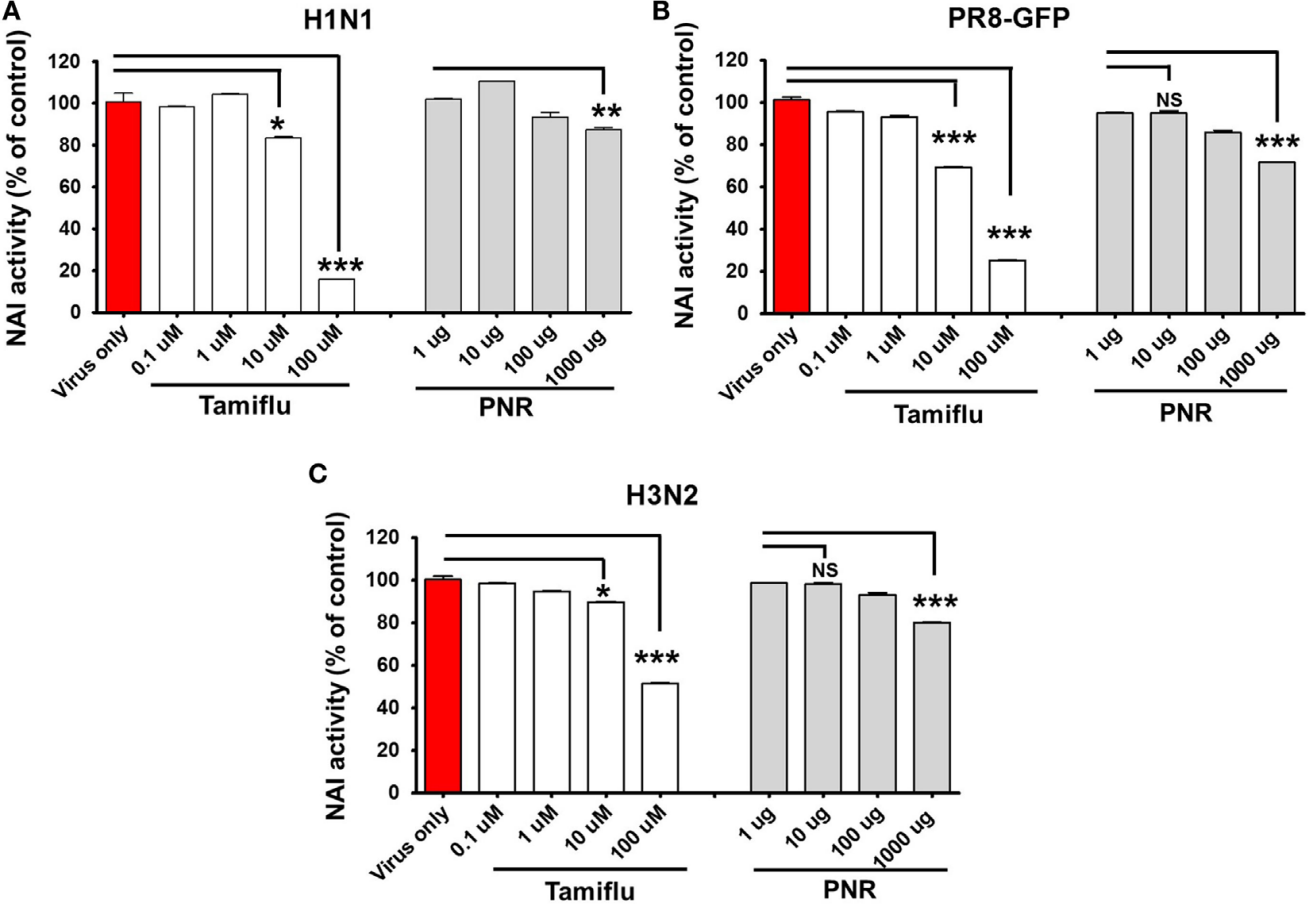
Measurement of the antiviral activity of *Panax notoginseng* root (PNR) using neuraminidase inhibition assays. Influenza A viruses (A/PR/8/34-GFP H1N1, and H3N2) were added at 32 hemagglutination units (HAUs) to the indicated concentrations of PNR, oseltamivir phosphate (positive control), or phosphate-buffered saline (PBS) (negative control), mixed with NA-Fluor™ substrate, and incubated at 37°C for 1 h in the dark **(A–C)**. Fluorescence was monitored *via* fluorescence spectrometry (excitation, 365 nm; emission, 445 nm). Data are presented as the mean ± SEM of three independent experiments. Bar graph (mean ± SEM) statistics were determined using two-way ANOVA with Bonferroni’s correction (posttest), ***P < 0.001; **P < 0.01; *P < 0.05. n.s., not significant.

We investigated whether PNR altered the NA activity of influenza A viruses. The activity of NA from A/PR/8/34, A/PR/8/34-GFP, and H3N2 was inhibited by PNR treatment in a concentration-dependent manner (Figure 7), However, only a high concentration of PNR (1,000 µg/mL) had suppressive effects on A/PR/8/34 (12.743%), A/PR/8/34-GFP (28.346%), and H3N2 (19.808%) NA.

### PNR Inhibited Influenza A Virus Infection in BALB/c Mice

**Figure 8:**
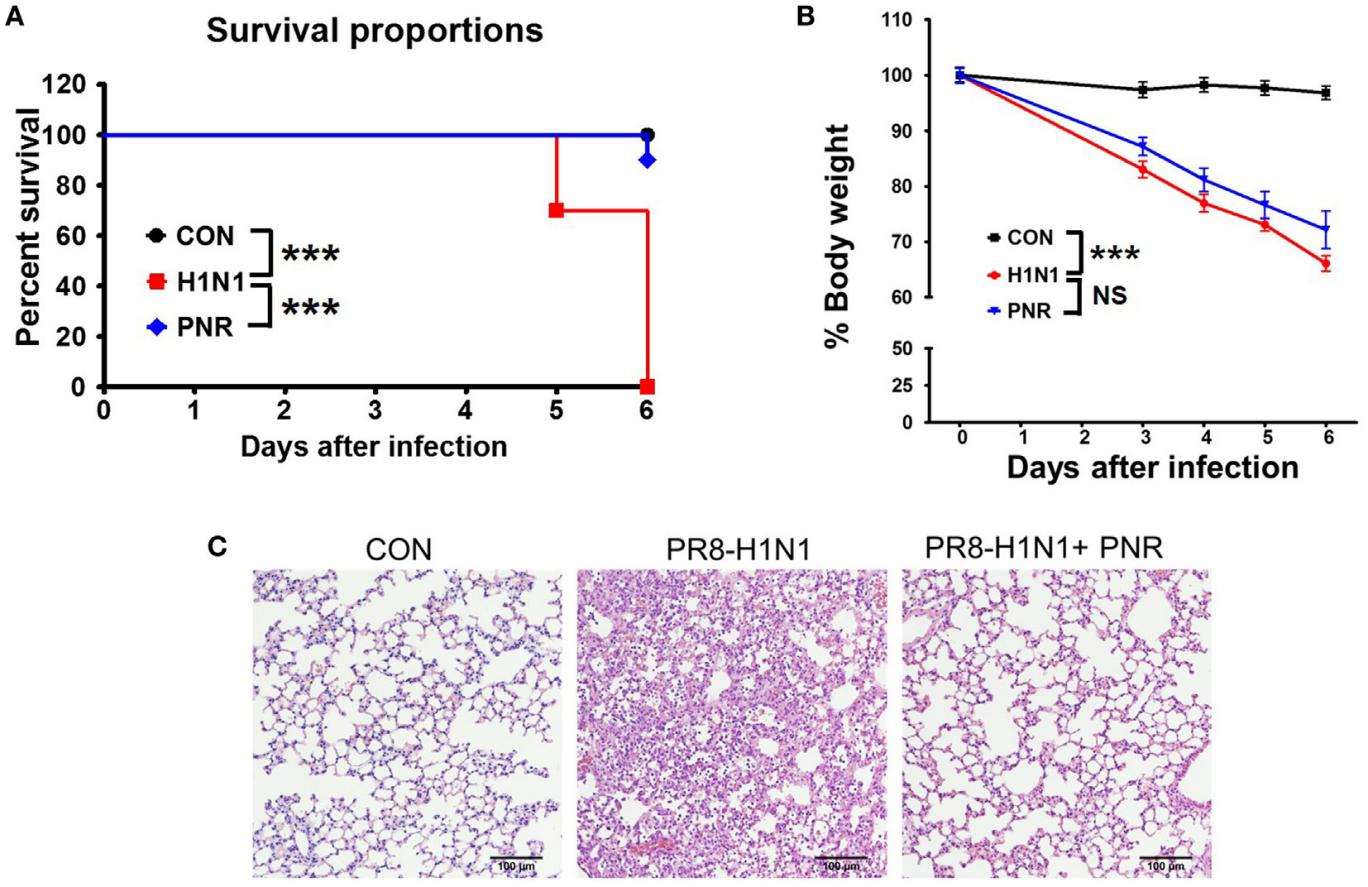
Effect of *Panax notoginseng* root (PNR) extract on influenza A virus infection in mice. BALB/c mice were pretreated orally with 100 mg/kg PNR (200 μL/mouse) water extract 1, 2, 3, 4, 5, 6, and 7 days before virus infection. **(A)** Percent survival and **(B)** body weight were monitored daily until 6 days post-infection. **(C)** A representative H&E image of histopathological damage in sectioned lung tissue from untreated mice or mice treated with PNR. To evaluate the significance of observed differences, the log-rank test **(A)** and two-way ANOVA with Bonferroni’s correction (posttest) **(B)** were used. ***P < 0.001, n.s., not significant. The mean and SEM are presented.

We first investigated the protective effects of PNR against influenza A virus infection in mice. Mice treated once daily with PNR (100 mg/kg) maintained a relatively stable body weight, and they did not exhibit any significant clinical symptoms throughout the study (data not shown). Untreated A/PR/8/34-infected mice exhibited significant body weight loss by 3–5 dpi before dying within 6 dpi (Figure 8). By contrast, PNR-treated mice displayed significantly decreased mortality and increased survival after A/PR/8/34 infection (Figure 8A). However, PNR (100 mg/kg) treatment did not protect against body weight loss following virus infection (Figure 8B). The lungs of untreated mice exhibited necrotic bronchial and bronchiolar epithelium, hemorrhage, alveolar thickening, and marked infiltration of inflammatory cells (Figure 8C). Conversely, PNR-treated mice displayed significantly decreased lung inflammation compared with the findings in untreated mice.

### Effects of PNR on NK Cell Activity of Isolated Splenocytes

**Figure 9:**
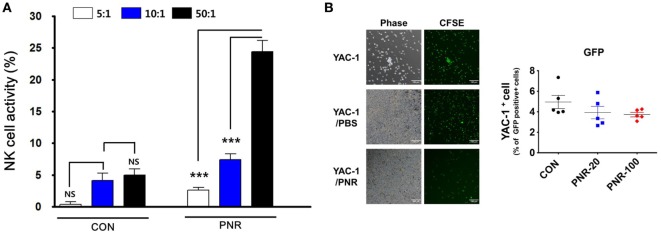
Natural killer (NK) cell activity in the splenocytes of mice administered *Panax notoginseng* root (PNR, 100 mg/kg) water extract. Splenocytes from PNR or phosphate-buffered saline (PBS)-administered mice (CON) were incubated with YAC-1 cells, and the released lactate dehydrogenase (LDH) content was determined *via* the LDH cytotoxicity assay or flow cytometric analysis and fluorescence microscopy. **(A)** NK cell activity in the splenocytes of mice at an effector cell:target cell ratio of 5:1, 10:1, or 50:1. **(B)** Spontaneous lysis of CFSE-labeled YAC-1 cells during incubation with splenocytes from PNR- or PBS-administered mice was compared with that in the absence of effector cells. Bar graph (mean ± SEM) statistics were determined using two-way ANOVA with Bonferroni’s correction (post-test). ***P < 0.001, n.s., not significant.

We investigated the effects of oral PNR (100 mg/kg) on NK cell activity using YAC-1 (target) cells and the LDH assay. At an effector cell:target cell ratio of 50:1, the NK cell activity of the splenocytes of PNR-treated mice was significantly increased compared with that of the splenocytes of PBS-treated mice (Figure 9A). In addition, we investigated NK cell activity and the cytotoxic effects of PNR on target cells *via* coculture with CFSE-labeled YAC-1 cells Figure 9B. After 24 h, as shown in Figure 9, splenocytes from PNR-administered mice displayed significantly enhanced NK cell activity against YAC-1 cells at an effector cell:target cell ratio of 50:1.

## Discussion

In humans and other organisms, the immune system is involved in maintaining homeostasis despite the constant barrage of attacks of infectious agents from the environment (52). Substantial research has focused on strengthening immune responses using active ingredients present in food and herbal medicines (53, 54).

The innate immune system, including components such as type-I IFN, is the first crucial line of host antiviral defense (55). In addition, type-I IFN plays a central role in antiviral responses by inducing the expression of antiviral genes that inhibit viral replication and induce apoptotic cell death in virally infected cells (56). The mechanism of innate antiviral immune responses mediated by pattern-recognition receptors (PRRs) has been a subject of intense investigation (57). PRRs including toll-like receptors, NOD-like receptors, DNA receptors, and RIG-I-like receptors recognize microbial components known as pathogen-associated molecular patterns, which lead to the production of type-I IFNs and pro-inflammatory cytokines (55, 58). PRRs require the key molecule TANK-binding kinase 1 (TBK1) to activate the transcription factor IRF3, which leads to type-I IFN induction and the expression of antiviral genes through the JAK/STAT pathway (59, 60).

Macrophages and NK cells classified as members of the immune system are known to play an important role in early defense against viral infections (30–32). Results have shown that the suppression of NK cells or their removal from mice can lead to morbidity and mortality, as well as delaying the removal of viruses after infection (33–36). NK cells are known to play a crucial role in innate immune responses to viruses such as influenza, particularly through cytotoxicity and cytokine release to infected target cells (31). Pro-inflammatory cytokines and IFNs that activate NK cells are activated by IFN or macrophage-derived cytokines and recognize the changes in MHC class-I expression, thus inhibiting the activation of uninfected cells and selectively inducing the apoptosis of infected cells.

Recent studies have shown that type-I IFN and STAT1 are required for NK cell responses following viral infection (37). In addition, type-I IFNs directly affect the expression of key activator molecules of NK cells through STAT1 phosphorylation (9). Thus, the generation of type-I IFNs and pro-inflammatory cytokines plays an important role in ensuring that the immune system can prevent NK cell activation and cytotoxicity (39–41). Studies have shown that immunomodulators increase host immunity, such as increasing NK cell activity and resistance to viral infections (42). In addition, IFNs such as IFN-β inhibit the replication of various viruses and function as key effector molecules in the immune response against viruses (61, 62). Thus, cytokines are promising targets for antiviral agents to enhance the host immune response to influenza virus infection. In addition, for rapid response to virus invasion, IFNs can upregulate specific immune response elements that can interfere with viral replication.

**Figure 10:**
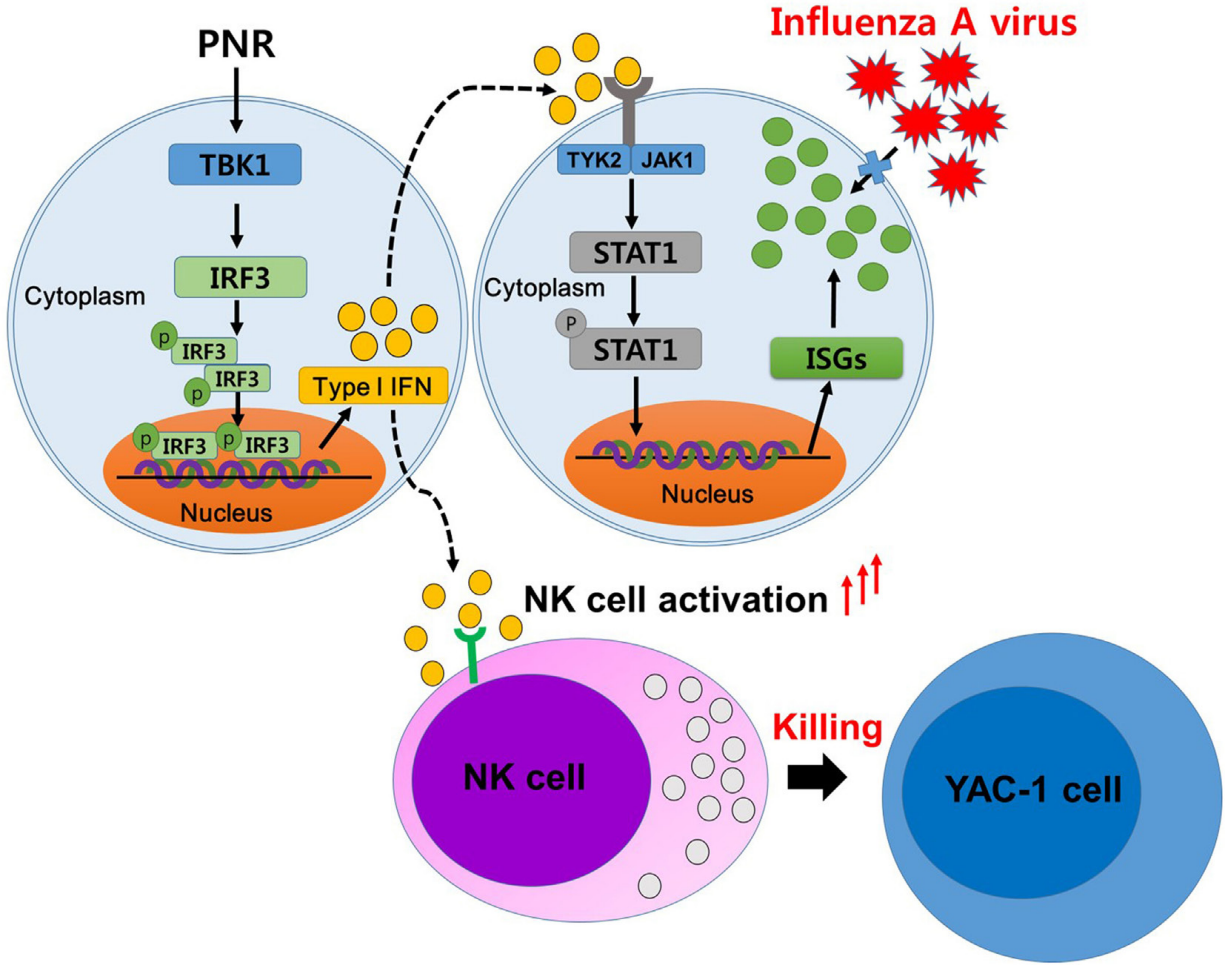
A schematic of the mechanism of *Panax notoginseng* root (PNR) in its enhancing of antiviral interferon-mediated immune responses and natural killer (NK) cell activity. PNR stimulates an antiviral state in murine macrophage cells by modulating the interferon (IFN) signaling pathway. PNR leads to the activation of IRF3 through TANK-binding kinase 1 (TBK1) and the production of type-I IFN. This secreted IFN can induce the inhibition of influenza A virus infection by stimulating the expression of stimulated target genes through the activation of signal transducer and activator of transcription 1, and these effects may be attributable to the ability of PNR to induce NK cell activity.

Against this background, many researchers have been studying herbs or natural products with immunomodulatory activity with the goal of overcoming influenza virus infection (15, 18, 43, 44). Thus, in this study, we performed a study on IRF3, TBK1, and STAT1 protein phosphorylation in PNR-treated RAW264.7 cells to determine the effect of PNR on the activation of type-I IFN signaling molecules. We confirmed that PNR significantly inhibited influenza virus infection in a dose-dependent manner in mouse macrophages. The findings also revealed that PNR pretreatment inhibited the expression of viral proteins and mRNAs. In the analysis of the mechanisms of action of PNR, PNR pretreatment was found to increase the production of IFN-β and pro-inflammatory cytokines (TNF-α and IL-6) and to induce the phosphorylation of type-I IFN-related proteins, such as TBK1, STAT1, and IRF3, *in vitro* (Figure 3). The results showed that type-I IFN binds to a component of the JAK/STAT pathway leading to the phosphorylation of STAT1 and transcriptional activation of the IFN-stimulated genes (51). Overall, the results suggest that PNR can induce an antiviral status of RAW264.7 cells by modulating the IFN signaling pathway, which can inhibit viral replication (Figure 10).

In line with this, PNR-treated mice exhibited a 90% reduction in mortality and protection against weight loss following H1N1 infection compared with the findings in untreated mice (Figure 8). This shows that the pretreatment of mice with PNR protected against the lethal effects of influenza A virus compared with the findings in the virus infection group without PNR. However, PNR showed a weak protective effect against the weight loss of mice at 9 days after influenza A virus infection. In influenza-infected mouse model, body weight decreases at an early stage of infection, but mice that survive after influenza virus infection show recovery of body weight and amelioration of infection-related symptoms. As such, in the case of PNR-pretreated mice that survived virus infection, they may also gain weight. Further, it is necessary to increase the duration of analysis in anti-influenza studies so as to find the factor underlying for PNR.

Major commercial ginseng is known as *P. ginseng* Meyer, *P. notoginseng*, and *P. quinquifolium* (63). Ginseng has been reported to be the most commonly used indication for treating indications, enhancing physical performance and having immunomodulatory activity (63). Chemical components of ginseng include pharmacological activities, such as ginsenosides, polysaccharides, and essential oils (64). Ginseng and its purified components possess protective effects against a wide range of viral infections (64–67). Notoginsenoside ST-4, isolated from *P. notoginseng*, inhibits penetration of herpes simplex virus (68). Recent studies have shown that the oligopeptide, a component of *P. ginseng*, modulates the innate adaptive immune response in mice by increasing phagocytic capacity and NK cell activity secretion in macrophages (69). In addition, ginsenoside Rb1 has enhanced natural killing activity of splenocytes (70) and ginsenoside Re also significantly enhanced serum-specific IgG in response to influenza A virus (71). In addition, the *P. notoginseng* saponins (72) and ginsenoside Rg3 (73) improved immune responses to OVA in mice. In this study, we also confirmed that PNR-administered mice displayed significantly enhanced NK cell activity against YAC-1 cells (Figure 9). If the above findings are taken together, PNR induces an antiviral state in murine macrophages and mice, leading to the inhibition of viral infection; these effects may be attributable to the ability of PNR to induce NK cell activity. Therefore, it can be seen that these constituent compounds, such as ginsenosides or polysaccharides, in the PNR synergistically increase the immune response. Thus, PNR has immunomodulatory effects on innate immune responses, providing potentially useful information for the application of prophylactic and therapeutic treatments for influenza A virus infection. However, in-depth studies are needed influenza A virus infection (Figure 10). The molecular mechanism of the immunomodulating effect of PNR and its components.

The swift emergence of new infectious viruses and drug-resistant variants has limited the availability of effective antiviral agents and vaccines. There is thus an urgent need to develop broad-spectrum antivirals and immunomodulatory agents that stimulate host immunity and improve host resilience. The antiviral activities of PNR are attributable to the enhancement of host immunity. Future studies should identify the components responsible for enhanced immunity against any viral attack. To determine the underlying molecular mechanisms associated with influenza infection, the antiviral activity of PNR components requires further investigation.

In summary, we demonstrated that PNR treatment decreased influenza A virus-induced mortality in mice by 90% and was protective against weight loss (by approximately 10%), compared with the findings in untreated mice. In addition, PNR administration increased the NK cell activity of mouse splenocytes. The protective effects of PNR components against influenza infection should be explored to obtain a deeper understanding of the underlying molecular mechanisms. In addition, on the basis of the obtained results, rational conclusions can be made regarding the antiviral effect of PNR in mouse models of infection. Taken together, these results suggest that PNR may be effective against infectious diseases such as influenza. On the basis of the results, we hypothesize that PNR is an effective antiviral agent or vaccine adjuvant for treating influenza virus infection.

## Ethics Statement

All animal experiments were carried out according to the guidelines for the care and use of laboratory animals of IACUC of the Laboratory Animal Center of DGMIF. The protocol was approved by the IACUC of DGMIF.

## Author Contributions

Developed the study design and revised the paper: JM, WC, and JC. Performed experiments: HL, YJ, NY, TO, and JC. Analyzed the data: JC and Y-HJ. Wrote the paper: JC.

## Conflict of Interest Statement

The authors declare that the research was conducted in the absence of any commercial or financial relationships that could be construed as a potential conflict of interest. The reviewer IM and handling editor declared their shared affiliation.

## Abbreviations

DGMIF, Daegu-Gyeongbuk Medical Innovation Foundation; ELISA, enzyme-linked immunosorbent assay; FBS, fetal bovine serum; GFP, green fluorescent protein; IACUC, Institutional Animal Care and Use Committee; KBPV, Korea Bank for Pathogenic Viruses; KIOM, Korea Institute of Oriental Medicine; KM, Korean medicine; NI, neuraminidase inhibition; NK, natural killer; PAMP, pathogen-associated molecular patterns; PBS, phosphate-buffered saline; PNR, *Panax notoginseng* root; PRR, pattern-recognition receptors; RLR, RIG-I-like receptors; UW, ultrapure water.
